# Supplementary material for: Monitoring high-intensity focused ultrasound thermal therapy by ultrasound doppler imaging using twinkling artifact
Source: PLoS One. 2025 Jul 2;20(7):e0324801. doi: 10.1371/journal.pone.0324801 (PMC12221069; doi:10.1371/journal.pone.0324801)
Supplement: Attachment — Submitted filename: Review.pdf [file pone.0324801.s001.pdf]

The paper titled *"Monitoring High-Intensity Focused Ultrasound (HIFU) Thermal Therapy by Ultrasound Doppler Imaging Using Twinkling Artifact"* explores an approach for monitoring HIFU-induced thermal lesions in tissue by leveraging the twinkling artifact (TA) in Doppler ultrasound. The study utilizes real-time Doppler imaging to detect frequency shifts caused by HIFU exposure in ex-vivo porcine tissue, creating visual markers of lesion formation. The findings indicate that TA in Doppler images can accurately monitor lesion dimensions during HIFU ablation with less than 10% error, presenting this method as a potentially effective alternative to MRI-guided HIFU monitoring. The approach's simplicity, cost-efficiency, and compatibility with existing ultrasound systems are highlighted as significant advantages over MRI and other ultrasound methods. There are several major concerns regarding this study, along with suggestions that could improve the quality of the paper.

Major Concerns and Suggestions:

1. The authors frequently use the term "novel" throughout the manuscript. However, there are already studies available that explore the use of the twinkling artifact in Doppler imaging to monitor HIFU, some of which are mentioned below. The authors should clearly elaborate on the novelty of their work compared to previous studies and ensure that all relevant prior research is cited. Additionally, I suggest that they avoid using the word "novel" and remove it from all sections of the manuscript.

   https://doi.org/10.1121/1.4800366
   DOI: 10.1109/TUFFC.2014.006502

2. As mentioned above as well, the introduction should focus on the work that has already been done in this field. All the available references in this field, including the most recent ones from 2024 on HIFU and HIFU monitoring, should be carefully added.
3. The methodology section requires further detail to improve reproducibility. Key aspects, such as the specific Doppler signal processing steps, frequency thresholds, and image construction parameters, need more explicit descriptions.
4. The manuscript omits important machine-specific parameters, such as Doppler frequency and pulse repetition settings, which could impact reproducibility across different systems.
5. While the ex-vivo results are promising, there is no in-vivo validation. Including a discussion on the limitations of ex-vivo results and plans for future in-vivo trials is necessary to account for physiological variations.
6. While the paper briefly compares Doppler TA with MRI, a more comprehensive analysis contrasting the proposed method with existing ultrasound-based techniques (e.g., elastography, local harmonic imaging) would strengthen the discussion. This could help emphasize its unique advantages or address comparable limitations.
7. The paper overlooks some technical challenges, such as variability in TA intensity across different depths and tissue types, which could impact clinical implementation. Including a discussion on how these factors might influence TA consistency and proposing solutions would enhance the study's practicality.
8. Beyond lesion size, additional quantitative assessments, such as boundary detection accuracy and frequency shift consistency, would help validate the method's precision. A quantitative error analysis of lesion boundary accuracy and TA intensity over time would further support the method's effectiveness and reliability.
9. Potential Doppler image artifacts, such as signal noise or low-intensity TA regions, are not addressed, which may impact lesion boundary detection.

10. The rationale behind selecting specific power levels (90W, 110W, 130W) is not detailed, potentially affecting reproducibility.
11. The study does not discuss how varying thermal doses impact TA visibility, potentially influencing lesion monitoring under different HIFU settings. The title of the paper explicitly mentions HIFU **thermal** therapy, implying that the thermal factor of HIFU would be the dominant factor in the therapy rather than the mechanical factor. However, there is no mention of the temperature increase amount due to each of the powers considered.
12. Potential clinical implementation challenges, such as patient movement and variability in tissue properties, are not discussed.
13. The study lacks a sensitivity analysis of Doppler TA detection, which would clarify the Doppler method's performance across different lesion sizes and depths.
14. In the end, while the idea of monitoring HIFU by using the twinkling artifact of ultrasound Doppler imaging is a quite interesting topic, the presentation of results is not sufficiently scientific, leading to issues with result interpretation. I suggest improving the discussion section with a more quantitative analysis of the obtained results. Additionally, mentioning the drawbacks of this work and suggesting possible study directions to address these technical issues would be beneficial as well.
