# Supplementary material for: Monitoring high-intensity focused ultrasound thermal therapy by ultrasound doppler imaging using twinkling artifact
Source: PLoS One. 2025 Jul 2;20(7):e0324801. doi: 10.1371/journal.pone.0324801 (PMC12221069; doi:10.1371/journal.pone.0324801)
Supplement: Attachment — Submitted filename: Response to Reviewers.docx [file pone.0324801.s004.docx]

We appreciate the reviewers' feedback. In this document, we respond to their comments. Additionally, we have provided two other separate files: one with the revised manuscript showing track changes, and another with the final revised manuscript.

Reviewer #1

1. The authors frequently use the term “novel” throughout the manuscript. However, there are already studies available that explore the use of the twinkling artifact in Doppler imaging to monitor HIFU, some of which are mentioned below. The authors should clearly elaborate on the novelty of their work compared to previous studies and ensure that all relevant prior research is cited. Additionally, I suggest that they avoid using the word “novel” and remove it from all sections of the manuscript.

Response: We ensured that the mentioned articles are properly cited in the revised manuscript. Additionally, we emphasized that the novelty of our work lies not in the general idea of using TA in HIFU therapy but in its specific application and findings.

2. As mentioned above as well, the introduction should focus on the work that has already been done in this field. All the available references in this field, including the most recent ones from 2024 on HIFU and HIFU monitoring, should be carefully added.

Response: New articles have been added to the introduction section to thoroughly cover prior work in this field.

3. The methodology section requires further detail to improve reproducibility. Key aspects, such as the specific Doppler signal processing steps, frequency thresholds, and image construction parameters, need more explicit descriptions.

Response: In response to the reviewer’s comment, we have added further details about the Doppler signal processing steps, frequency thresholds, and constructed image parameters in the revised manuscript. Specifically, the dataset used contains back-scattered RF signals from samples, from which Doppler signals were extracted using the method described in the methodology section. This method can process signals containing all samples in each time series (80 samples), but we opted to calculate frequency features in segmented time signals to enable real-time reporting and guide HIFU treatment effectively.

We tested segments of 1 to 10 samples and ultimately decided on 10-sample segments with a one-sample step size and nine-sample overlap to reduce computational load. FFT was then applied to these signals to obtain the phase and amplitude of each segment. Next, the maximum phase of each signal was extracted, and the phase and corresponding amplitude were stored for each pixel.

To extract frequency from the phases, we first removed noise effects by applying a threshold to the amplitudes, set at one-tenth of the maximum amplitude. This threshold ratio was determined through several trials. Frequencies were then extracted by subtracting the phase of each pixel in two consecutive images. These frequencies represent the Doppler shift frequency, and by displaying the matrices of these extracted frequencies, we generated the Doppler images.

4. The manuscript omits important machine-specific parameters, such as Doppler frequency and pulse repetition settings, which could impact reproducibility across different systems.

Response: In the experiment underlying this study, the back-scattered signals and B-mode images were recorded, and the Doppler frequency shift was extracted using the method described in the methodology section.

5. While the ex-vivo results are promising, there is no in-vivo validation. Including a discussion on the limitations of ex-vivo results and plans for future in-vivo trials is necessary to account for physiological variations.

Response: We acknowledge the reviewer's concern regarding the lack of in-vivo validation in our study. In the revised manuscript, we included a discussion on the limitations of ex-vivo results and outline plans for future in-vivo trials to account for physiological variations. Specifically, we emphasized that for accurate calculations, it is preferable to use this method in stationary tissues and avoid areas with physiological movement, such as the heart and lungs.

6. While the paper briefly compares Doppler TA with MRI, a more comprehensive analysis contrasting the proposed method with existing ultrasound-based techniques (e.g., elastography, local harmonic imaging) would strengthen the discussion. This could help emphasize its unique advantages or address comparable limitations.

Response: In the discussion section, we have already addressed the advantages and disadvantages of other ultrasound-based methods. Given the need for further clarification, we edited this section and added a more precise comparison and discussion of the developed method in relation to other ultrasound-based techniques.

7. The paper overlooks some technical challenges, such as variability in TA intensity across different depths and tissue types, which could impact clinical implementation. Including a discussion on how these factors might influence TA consistency and proposing solutions would enhance the study’s practicality.

Response: In the discussion section, we mentioned that future studies on investigating influencing factors on the twinkling artifact could be beneficial. However, given that the twinkling artifact is observed with changes in the phase of the signal, we noted that while the amplitude vary with depth, the phase, as far as we know, remains unchanged. As a result, changes in depth are not expected to affect the twinkling artifact.

8. Beyond lesion size, additional quantitative assessments, such as boundary detection accuracy and frequency shift consistency, would help validate the method's precision. A quantitative error analysis of lesion boundary accuracy and TA intensity over time would further support the method's effectiveness and reliability.

Response: We agree that assessing boundary detection accuracy and frequency shift consistency could further validate the precision of the proposed method. Additionally, analyzing TA intensity over time would strengthen the method's effectiveness and reliability. However, there are limitations in the available data. For this study, only photographs of sliced samples taken long after HIFU treatment were available, which prevented real-time analysis during treatment.

In future studies, our group plans to use MRI and Doppler ultrasound separately but simultaneously. This approach would allow MRI to provide high accuracy in detecting lesion dimensions and boundaries, while Doppler ultrasound would enable precise tracking of twinkling artifacts (TAs) using both research and commercial devices. By using both methods in parallel, we can perform a quantitative error analysis of lesion dimensions, boundary detection, and TA intensity during treatment, leading to a more effective and reliable method.

9. Potential Doppler image artifacts, such as signal noise or low-intensity TA regions, are not addressed, which may impact lesion boundary detection.

Response: According to the literature, the twinkling artifact has been observed in the imaging of kidney and urinary tract stones. To our knowledge, there are no reports of this artifact occurring in soft tissues under HIFU. Signals exhibiting the twinkling artifact involve severe phase shifts. Given the strong agreement between the lesion sizes in the constructed images and the dimensions observed in the cut specimens, it does not appear that weak amplitudes significantly impact the measurements. Additionally, these findings were validated at different stages, further confirming the reliability of the results.

10. The rationale behind selecting specific power levels (90W, 110W, 130W) is not detailed, potentially affecting reproducibility.

Response: The experiment underlying this study was conducted at an earlier stage, where coagulative necrosis was observed at higher power levels. Based on these findings, the power levels of 90W, 110W, and 130W were selected to investigate the developed method.

11. The study does not discuss how varying thermal doses impact TA visibility, potentially influencing lesion monitoring under different HIFU settings. The title of the paper explicitly mentions HIFU thermal therapy, implying that the thermal factor of HIFU would be the dominant factor in the therapy rather than the mechanical factor. However, there is no mention of the temperature increase amount due to each of the powers considered.

Response: This study focuses on developing an ultrasound-based image guidance method for monitoring HIFU thermal lesion location. While thermometry is undoubtedly important in the treatment process, this manuscript emphasizes local guidance of the treatment. Previous papers from our laboratory and by the authors of this study have explored thermometry of thermal lesions, and we mentioned that thermometry could be nenfical in lesion monitoring in the discussion section in the revised manuscript.

12. Potential clinical implementation challenges, such as patient movement and variability in tissue properties, are not discussed.

Response: The limitations of using ultrasound-guided systems (including the developed method) for HIFU therapy, including variations in tissue properties and patient movement, addressed in the Discussion section of the revised manuscript.

13. The study lacks a sensitivity analysis of Doppler TA detection, which would clarify the Doppler method’s performance across different lesion sizes and depths.

Response: The primary focus of this study was on the presence of twinkling artifacts (TAs) in thermal lesions induced by HIFU therapy. Based on current understanding, TAs originate from phase changes in backscattered signals. Since, to the best of our knowledge, these phase changes are not significantly affected by depth or tissue properties, it can be inferred that depth and tissue type have minimal impact on the overall presence of TAs.

14. In the end, while the idea of monitoring HIFU by using the twinkling artifact of ultrasound Doppler imaging is a quite interesting topic, the presentation of results is not sufficiently scientific, leading to issues with result interpretation. I suggest improving the discussion section with a more quantitative analysis of the obtained results. Additionally, mentioning the drawbacks of this work and suggesting possible study directions to address these technical issues would be beneficial as well.

Response: We sincerely appreciate your thoughtful feedback on this study. The Discussion and Results sections have improved to include a more quantitative analysis of the results. Additionally, the drawbacks of this work and potential future directions to address these technical challenges were discussed. A comparison of the proposed method with other existing methods included to provide a more comprehensive perspective.

Reviewer #2:

Major Comments:

Methodology and Experimental Design:

The manuscript lacks sufficient methodological details, particularly regarding the Doppler signal processing steps. Essential steps such as noise reduction techniques and parameter settings for Fast Fourier Transform (FFT) are vaguely described, which may hinder reproducibility.

The rationale for choosing specific parameters, such as sampling frequency and Doppler imaging intervals, is not clearly justified. Clarification is essential for verifying the robustness of the technique under different settings.

The study's experimental design relies on ex-vivo porcine tissue samples, which limit its clinical relevance. Extending the work to in-vivo studies is essential to validate the method for real-time HIFU lesion monitoring in a clinical context.

Response: Methodology and Experimental Design:

Doppler Signal Processing Details:

In this study, backscattered signals were utilized, and Doppler signals were not directly recorded. As outlined in the Methods section, Doppler signals were extracted from the backscattered signals using the approach described in the book by Cobbold. To omit noise with low amplitudes but significant phase changes, we applied a noise reduction step. Specifically, amplitudes less than one-tenth of the maximum amplitude were removed. This threshold was determined by evaluating several different thresholds and selecting the optimal value. For samples with amplitudes below this threshold, the phase value was set to zero, while the phase values of samples above the threshold were preserved. The phase changes between consecutive acquisitions were then used to calculate the frequency shift.

FFT Parameter Settings and Real-Time Processing: To ensure real-time reporting while maintaining computational efficiency, we implemented a processing strategy where every 10 samples were used to calculate the Fast Fourier Transform (FFT). This approach involved a one-sample step forward with 9 overlapping samples, balancing high accuracy in frequency content reporting with reduced computational load.

Clinical Relevance and In-Vivo Validation: We agree that in-vivo validation is essential for clinical relevance. In the Discussion section, we have noted the importance of validating this method in in-vivo experiments.

Data Analysis and Interpretation:

The authors report a less than 10% error in depth and width measurements for coagulated tissue dimensions; however, statistical validation is missing. Including statistical metrics, such as confidence intervals or p-values, would strengthen the claim.

The frequency analysis and Doppler image construction methods are introduced but not supported with quantitative analyses of accuracy or precision compared to established methods.

The constructed Doppler images (Figure 5) require more clarity, especially regarding the artifacts that are intended to represent TAs. The images should be annotated and analyzed with better-defined metrics.

Response: Data Analysis and Interpretation:

In the revised manuscript, statistical validation, including p-values, provided to support the reported error rates for depth and width measurements of coagulated tissue dimensions. The method used in this study was selected based on the limitations of the available data. The primary focus was on investigating the presence or absence of twinkling artifacts (TAs) in the images, which is why quantitative accuracy or precision analyses were not initially included. We have clarified the Doppler images (Figure 5) captions.

Technical Limitations and Applicability:

Despite discussing the limitations of existing MRI and ultrasound-based methods, the manuscript does not provide a comprehensive comparison of how the proposed Doppler imaging approach surpasses or falls short of these alternatives. Explicit benchmarks or performance comparisons would be beneficial.

The proposed method’s sensitivity and specificity in detecting TAs related to HIFU lesions are not adequately evaluated, limiting the manuscript's practical value in guiding clinical applications.

Response: Technical Limitations and Applicability:

A comprehensive comparison between the proposed Doppler imaging approach and existing MRI and ultrasound-based methods added in the revised manuscript.

Lack of Innovation and Literature Gap:

Although the use of Doppler imaging to monitor HIFU treatment is potentially valuable, the manuscript does not sufficiently differentiate itself from prior works on TAs in sonography. The novelty of the approach is undermined by a lack of unique insights or innovative techniques. A more thorough literature review that establishes a genuine gap is necessary.

Response: Lack of Innovation and Literature Gap:

The primary goal of this study was to investigate and develop a method to enable the use of HIFU therapy. The literature review highlights the current limitations in monitoring HIFU treatments. Additionally, the Discussion section emphasizes that the advantages of the proposed method, once validated in in-vivo studies, could significantly expand the applicability of HIFU therapy. The investigation of twinkling artifacts (TAs) in soft tissues undergoing coagulative necrosis due to HIFU represents a novel aspect of this work. However, we have enhanced the literature review to provide a more comprehensive summary of the research gap and clearly articulate the significance and novelty of this study.

Ethics and Data Availability:

There is no explicit ethical consideration or discussion on data accessibility. Given that the study could have implications for human subjects in future clinical applications, ethical considerations need to be addressed. Additionally, data availability should be transparent to ensure reproducibility.

Response: Ethics and Data Availability:

The animal tissue used in the experiments for this study was purchased from a local butchery. All experiments were conducted on ex-vivo tissues following the procedures outlined in Rangraz et al., 2014. Ethical considerations for future clinical applications involving human subjects addressed in the revised manuscript. Additionally, data accessibility clarified to ensure reproducibility.

Minor Comments:

The manuscript has several typographical and grammatical errors, which detract from the readability and professional presentation. A thorough proofreading is recommended.

Figures, particularly 5 and 6, lack sufficient annotations, which makes interpreting the results challenging. Including color-coded legends and scale bars would improve clarity.

The reference section includes several incomplete citations, with missing links to data sources and studies.

Minor Comments:

Proofreading: The revised manuscript have been checked to correct typographical and grammatical errors.

Figure Annotations: the captions of figures 5 and 6 have been edited.

References: The reference section has been be reviewed and updated to ensure completeness, including proper links to data sources and studies.
