# Supplementary material for: Monitoring high-intensity focused ultrasound thermal therapy by ultrasound doppler imaging using twinkling artifact
Source: PLoS One. 2025 Jul 2;20(7):e0324801. doi: 10.1371/journal.pone.0324801 (PMC12221069; doi:10.1371/journal.pone.0324801)
Supplement: Attachment — Submitted filename: Review Comments.docx [file pone.0324801.s003.docx]

I thank the authors for addressing the comments. Most of the concerns have been answered sufficiently, but some issues remain regarding the responses. Additionally, some technical refinements are necessary in the manuscript.

**Unaddressed or Incompletely Addressed Points**

1. **Justification for Parameter Selection:** The justification for choosing specific FFT parameters, noise thresholds, and segmentation window sizes is not detailed. The explanation of Doppler signal extraction and processing could be expanded. Specifically, the choice of a 10-sample window and the noise thresholding method require additional justification. Were these parameters optimized through testing or based on prior literature?
2. **Quantitative Comparison of Doppler TA to Other Modalities:** While the authors compared Doppler TA to MRI and ultrasound elastography, a **quantitative** comparison (e.g., sensitivity, specificity, accuracy) is lacking. The discussion on alternative imaging modalities (MRI, elastography, etc.) is useful, but quantitative performance comparisons (e.g., sensitivity/specificity, resolution) would strengthen the argument for Doppler TA’s clinical relevance.
3. **Figure Modifications:** Some figures (e.g., Figures 5 and 6) were updated based on reviewer comments but still need modification. The color bar explanation should not be only in the caption. The figure should be self-explanatory, allowing readers to grasp all relevant information at a glance. The color bar lacks a title indicating what the colors represent. Although mentioned in the caption, the figure itself should clearly display this information. Please add a title to the color bar in each of the figures.
4. **Statistical Validation Details:** Although standard deviation (SD) and confidence intervals were added, a more detailed explanation of the statistical methods used for lesion measurement validation is missing. Further details on how SD and confidence intervals were calculated would improve transparency. Additionally, were interobserver variability or repeatability tests performed?

**Minor Technical Refinements**

1. **Terminology Consistency:** There are inconsistencies in the use of terms such as "slow-time signals," "fast-time signals," and "RF signals." Ensure that terminology is uniform throughout the manuscript.
2. **Equation Numbering and Formatting:** Some equations (e.g., Doppler shift formula) are not properly referenced within the text. Ensure that equations are numbered correctly and referred to in explanations.
3. **Language and Grammar Check:** Although the authors claim that proofreading was done, it does not appear to have been performed by a native English speaker, as many minor grammatical errors remain. A final proofread for clarity and conciseness is essential. Some examples of errors are as follows:

- **Missing Articles ("a," "an," "the")**

**Example 1:** "The Twinkling Artifact (TA) is color Doppler artifact caused by acoustic radiation force and consequent tissue vibration during Doppler imaging."

**Correction:** "The Twinkling Artifact (TA) is a color Doppler artifact caused by the acoustic radiation force and the consequent tissue vibration during Doppler imaging."

**Issue:** "A" was missing before "color Doppler artifact," and definite articles ("the") were needed.

**Example 2:** "Figure 5 and 6 could benefit from clearer annotations."

**Correction:** "Figures 5 and 6 could benefit from clearer annotations."

**Issue:** When referring to multiple figures, "Figures" (plural) should be used instead of "Figure."

- **Subject-Verb Agreement**

**Example 1:** "The Doppler images that was constructed show the lesion formation."

**Correction:** "The Doppler images that were constructed show the lesion formation."

**Issue:** "Images" is plural, so the verb should be "were" instead of "was."

**Example 2:** "These results suggest that Twinkling Artifact is useful in monitoring thermal lesions and are in agreement with previous studies."

**Correction:** "These results suggest that the Twinkling Artifact is useful in monitoring thermal lesions and is in agreement with previous studies."

**Issue:** "Twinkling Artifact" is singular, so it should be "is" instead of "are."

- **Inconsistent Tense Usage**

**Example 1:** "In the study, we show that the Doppler signal extraction method provides reliable results and confirmed the presence of TA."

**Correction:** "In this study, we show that the Doppler signal extraction method provides reliable results and confirm the presence of TA."

**Issue:** The sentence shifts between present ("show") and past ("confirmed")—both should be in present tense for consistency.

**Example 2:** "The proposed method was tested and demonstrates a high level of accuracy."

**Correction:** "The proposed method was tested and demonstrated a high level of accuracy."

**Issue:** "Was tested" is past tense, so "demonstrates" should also be changed to past tense ("demonstrated") for consistency.

- **Clarity and Redundancy**

**Example 1:** "This study aims to investigate and analyze the role of the Twinkling Artifact for the purpose of lesion monitoring."

**Correction:** "This study analyzes the role of the Twinkling Artifact in lesion monitoring."

**Issue:** "Investigate and analyze" are redundant, and "for the purpose of" can be replaced with a simpler "in."

**Example 2:** "The obtained results are consistent and in agreement with previous studies."

**Correction:** "The results are consistent with previous studies."

**Issue:** "Consistent" and "in agreement" mean the same thing—one should be removed.

There are many many more issues regarding awkward phrasing and wordiness. Reading this manuscript is difficult due to the overwhelming number of grammatical errors. A simple grammar checker, such as **Grammarly**, would eliminate most of these issues!!!
