# Supplementary material for: Monitoring high-intensity focused ultrasound thermal therapy by ultrasound doppler imaging using twinkling artifact
Source: PLoS One. 2025 Jul 2;20(7):e0324801. doi: 10.1371/journal.pone.0324801 (PMC12221069; doi:10.1371/journal.pone.0324801)
Supplement: Attachment — Submitted filename: Response_to_Reviewers_auresp_2.docx [file pone.0324801.s005.docx]

We appreciate the reviewers' feedback. In this document, we respond to their comments. Additionally, we have provided two other separate files: one with the revised manuscript showing track changes, and another with the final revised manuscript.

Reviewer #1

1. Justification for Parameter Selection: The justification for choosing specific FFT parameters, noise thresholds, and segmentation window sizes is not detailed. The explanation of Doppler signal extraction and processing could be expanded. Specifically, the choice of a 10-sample window and the noise thresholding method require additional justification. Were these parameters optimized through testing or based on prior literature?

Response: As noted in our prior response, the 10-sample window and noise threshold (1/10th max amplitude) were empirically optimized through systematic testing. These parameters balanced real-time processing needs with signal fidelity during HIFU guidance. We have expanded the explanation of Doppler signal extraction in the revised manuscript's methodology section.

2. Quantitative Comparison of Doppler TA to Other Modalities: While the authors compared Doppler TA to MRI and ultrasound elastography, a quantitative comparison (e.g., sensitivity, specificity, accuracy) is lacking. The discussion on alternative imaging modalities (MRI, elastography, etc.) is useful, but quantitative performance comparisons (e.g., sensitivity/specificity, resolution) would strengthen the argument for Doppler TA’s clinical relevance.

Response: We appreciate this valuable suggestion regarding quantitative comparisons. While our current study focused primarily on demonstrating the feasibility of Doppler TA for thermal lesion monitoring, we fully acknowledge that direct quantitative comparisons with MRI and ultrasound elastography would strengthen clinical relevance.

As noted in our future work section, we are currently designing follow-up studies involving simultaneous MRI and Doppler ultrasound acquisitions to enable rigorous, head-to-head performance comparisons (including sensitivity/specificity metrics) under controlled conditions. These planned experiments will specifically address the important comparative metrics the reviewer has highlighted.

3. Figure Modifications: Some figures (e.g., Figures 5 and 6) were updated based on reviewer comments but still need modification. The color bar explanation should not be only in the caption. The figure should be self-explanatory, allowing readers to grasp all relevant information at a glance. The color bar lacks a title indicating what the colors represent. Although mentioned in the caption, the figure itself should clearly display this information. Please add a title to the color bar in each of the figures.

Response: We have added the requested color bar titles to Figures 5 and 6 in the new revised manuscript.

4. Statistical Validation Details: Although standard deviation (SD) and confidence intervals were added, a more detailed explanation of the statistical methods used for lesion measurement validation is missing. Further details on how SD and confidence intervals were calculated would improve transparency. Additionally, were interobserver variability or repeatability tests performed?

Response: For the added statistical metrics, we utilized measurements derived from different frame sequences. As detailed in the Data and Methods section, our dataset consisted of:

- 16 pre-HIFU frames
- 40 frames during HIFU sonication
- 9 immediate post-HIFU frames
- 16 frames acquired 10 minutes post-HIFU

Since the histological samples were obtained long after HIFU treatment and quantitative measurements of actual lesion dimensions during treatment were unavailable, we compared:

- Final lesion dimensions from histological sections
- Dimensions measured from the last 16 Doppler image frames
  This comparative analysis enabled us to calculate and include the statistical metrics (standard deviation and confidence intervals).

A key objective of our future studies involves implementing simultaneous MRI monitoring, which will allow real-time evaluation of our developed method during treatment procedures.

Minor Technical Refinements

1.Terminology Consistency: There are inconsistencies in the use of terms such as "slow-time signals," "fast-time signals," and "RF signals." Ensure that terminology is uniform throughout the manuscript.

Response: Done!

2. Equation Numbering and Formatting: Some equations (e.g., Doppler shift formula) are not properly referenced within the text. Ensure that equations are numbered correctly and referred to in explanations.

Response: Done!

3. Language and Grammar Check: Although the authors claim that proofreading was done, it does not appear to have been performed by a native English speaker, as many minor grammatical errors remain. A final proofread for clarity and conciseness is essential. Some examples of errors are as follows:
Response: Done!
